# Pneumatosis intestinalis post steroid use in a patient with immune-related adverse events: Case report, literature review and FAERS analysis

**DOI:** 10.3389/fphar.2023.1133551

**Published:** 2023-03-14

**Authors:** Tingting Zhang, Mingnan Cao, Bin Zhao, Chen Pan, Li Lin, Chuanhao Tang, Zhigang Zhao, Jingli Duan, Li Wang, Jun Liang

**Affiliations:** ^1^ Department of Clinical Oncology, Peking University International Hospital, Beijing, China; ^2^ Department of Pharmacy, Beijing Tiantan Hospital, Capital Medical University, Beijing, China; ^3^ Department of Pharmacy, Peking Union Medical College Hospital, Peking Union Medical College, Chinese Academy of Medical Sciences, Beijing, China; ^4^ Department of Pharmacy, Beijing Friendship Hospital, Capital Medical University, Beijing, China; ^5^ Department of Pharmacy, Peking University International Hospital, Beijing, China; ^6^ Clinical Trial Center, Peking University International Hospital, Beijing, China

**Keywords:** pneumatosis intestinalis, steroid, lung carcinoma, immune-related adverse events, FAERS, immune checkpoint inhibitor

## Abstract

**Introduction:** The accurate diagnosis of pneumatosis intestinalis (PI) is increasing despite patients’ limited identification of etiologic factors. Recently a patient with lung squamous carcinoma who developed pneumatosis intestinalis following methylprednisolone administration for immune-related adverse events was treated at our hospital. Subsequent a literature review and an analysis of the FDA Adverse Event Reporting System (FAERS) database enabled the identification of additional cases of pneumatosis intestinalis.

**Methods:** A literature review of the MEDLINE/PubMed and Web of Science Core Collection databases using standard pneumatosis intestinalis search terms to identify published cases of immune checkpoint inhibitors (ICIs) or steroids causing pneumatosis intestinalis were performed. A separate retrospective pharmacovigilance study of FAERS enabled the extraction of unpublished cases of pneumatosis intestinalis between the first quarter of 2005 and the third quarter of 2022. Disproportionality and Bayesian analyses were performed to identify signal detection in reported odds ratios, proportional reporting ratios, information components, and empirical Bayesian geometric means.

**Results:** Ten case reports of steroid-related pneumatosis intestinalis were retrieved from six published studies. The implicated drug therapies included pre-treatment with steroids before chemotherapy, combination therapy with cytotoxic agents and steroids, and monotherapy with steroids. In the FAERS pharmacovigilance study, 1,272 cases of immune checkpoint inhibitors or steroid-related pneumatosis intestinalis were incidentally reported. The signal detected in five kinds of immune checkpoint inhibitors and six kinds of steroids implied a positive correlation between the drugs and adverse events.

**Conclusion:** Steroids might be the etiologic factors in the current case of pneumatosis intestinalis. Reports supporting the role of steroids in suspected cases of pneumatosis intestinalis can be found in literature databases and the FAERS database. Even so, as documented in FAERS, immune checkpoint inhibitors-induced pneumatosis intestinalis should not be excluded.

## 1 Introduction

Pneumatosis intestinalis (PI) is an uncommon condition characterized by accumulating radiologically detected submucosal or subserosa gas cysts in the gastrointestinal wall (Heng et al., 1995). PI is associated with severe life-threatening complications. Clinical manifestations of PI range from asymptomatic to fatal, and its symptomatology includes abdominal pain, abdominal distention, nausea, vomiting, diarrhea, and constipation (Wang et al., 2018; Ling et al., 2019). Etiological factors of PI include intestinal diseases, systemic diseases, pulmonary diseases, medications, iatrogenic causes, and trauma (Lee and Wu, 2019).

Recently, a patient presented to our hospital with squamous lung carcinoma and PI secondary to prednisone use due to immune-related adverse events (irAEs). Accurate diagnosis of PI prevents unnecessary abdominal surgeries. In addition, immune checkpoint inhibitors (ICIs) increase patients’ immunities. Consequently, clinicians are increasingly confronted with irAEs requiring steroid management. Therefore, we performed a literature search for published cases of PI associated with ICIs or steroids. In addition, we reviewed the FDA Adverse Event Reporting System (FAERS) database to identify additional cases of steroids-induced or ICIs-induced PI.

## 2 Case description

A 62-year-old male smoker with a 20-pack-year smoking history was admitted to the Department of Clinical Oncology at Peking University International Hospital complaining of hemoptysis in November 2020. Positron emission tomography/computed tomography (CT) revealed a 21 mm × 25 mm mass on the superior lobe of the right lung with enlarged mediastinal, bilateral hilar, and right supraclavicular lymph nodes. Further histopathological and molecular testing confirmed the diagnosis of squamous lung carcinoma in the absence of driver mutations. Immunohistochemical staining showed programmed cell death 1 ligand 1 (PD-L1) expression in 40% of the tumors. Standard platinum-based chemotherapy, including paclitaxel liposomes and carboplatin, was initiated. Contrast-enhanced CT scans revealed no responses after two cycles. According to the multidisciplinary team, the patient received two doses of 200 mg sintilimab in a three-week cycle with concurrent standard platinum-based chemotherapy. CT evaluation showed a partial response, with approximately 80% reduction in the size of the primary pulmonary lesions. The patient underwent definitive 60 Gy of thoracic radiotherapy with standard fractionation (2 Gy/fraction) between 4 March 2021, and 7 April 2021. Radiological evaluation revealed durable clinical responses. Subsequently, sintilimab treatment as consolidation was started 5 weeks after completing a 3-week course of radiotherapy.

After the third cycle of sinitilimab, the patient experienced dizziness, fatigue, nausea, and loss of appetite. Endocrinological examinations and brain Magnetic Resonance Imaging results suggested combined hypothyroidism and secondary adrenocortical insufficiency induced by sinitilimab. Hormone replacement therapy was administered, including physiological replacement doses of glucocorticoids and thyroxine. The patient’s symptoms disappeared rapidly, and laboratory data spontaneously improved. Two months later, the fourth sintilimab infusion was administered. On 28 October 2021, total body CT showed continued partial response, with new consolidation. Based on multidisciplinary team, pneumonitis was diagnosed as a mild form of grade II (according to CTCAE 4.0). In addition to sintilimab discontinuation, systemic high-dose glucocorticoid therapy was prescribed (60 mg intravenous methylprednisolone daily for 7 days, followed by 40 mg oral methylprednisolone daily for 7 days, tapered gradually). Repeat CT showed improvement after 4 weeks, without tumor progression.

**FIGURE 1:**
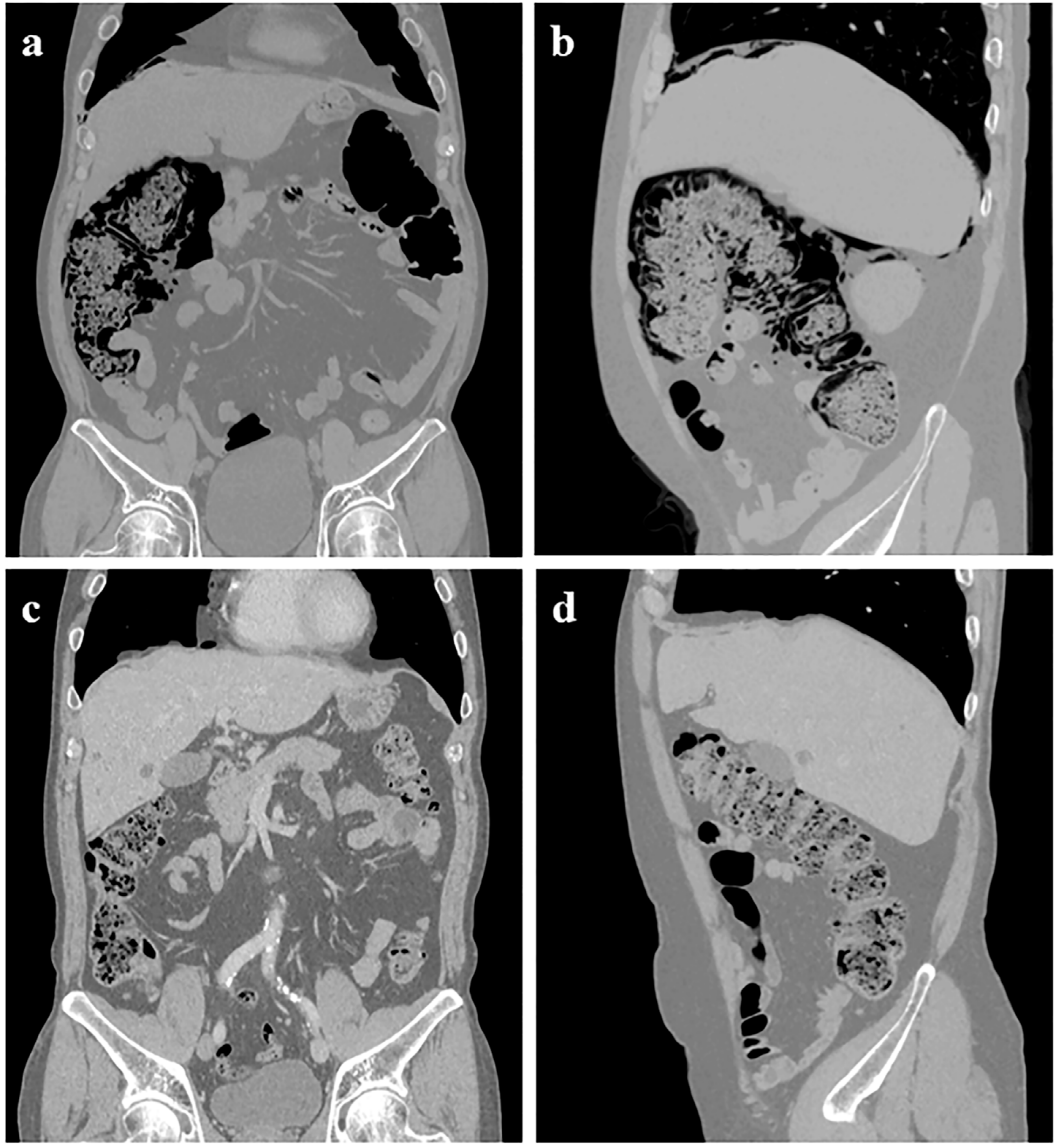
Abdominal CT showed pneumoperitoneum under the right diaphragm, extensive intramural air in the ascending colon, and hepatic flexure with massive air accumulation in the colon 6 weeks after starting high-dose methylprednisolone **(A–B)**. However, these findings had almost completely disappeared on abdominal CT taken at the outpatient clinic **(C–D)**.

Unfortunately, the patient was admitted to our hospital due to progressive abdominal distension for 2 weeks on 17 December 2021. His vital signs were stable on arrival. However, a physical examination revealed hypoactive bowel sounds. Although the abdomen was non-tender with a tympanic percussion note. Abdominal X-Ray and CT examination suggested massive gas accumulation in the right half of the colon, gas in the intestinal wall, and free air under the diaphragm (Figures 1A, B). The patient was diagnosed with pneumoperitoneum and PI. Leukocytosis or C-reactive protein elevation was absent on blood film examination. However, renal function, liver function, and electrolytes, including potassium and sodium, were normal. Endocrinological examination revealed normal thyroid function, and serum cortisol and adreno cortico tropic hormone levels were within the lower limit. Physiological Hormone replacements with levothyroxine and prednisone acetate were administered daily to treat hypothyroidism and secondary adrenocortical insufficiency. Conservative management was recommended after a consultation between a gastrointestinal surgeon and a gastroenterologist. Parenteral nutrition, gastrointestinal decompression, and oxygen inhalation (3 mL/min) were initiated, and oral antibiotics (metronidazole) were used for therapeutic purposes. Flatulence and abdominal distention improved after treatment was initiated. CT showed reduced findings of gas in the abdomen, and the patient resumed a normal diet before discharge (Figures 1C, D). After sintilimab discontinuation, CT revealed a durable clinical response with residual actinic fibrosis. The patient has been in excellent condition without further anti-cancer therapies and immunotherapy for approximately a year.

## 3 Literature review of steroid-related PI cases

### 3.1 Methods

A literature review of the MEDLINE/PubMed and Web of Science Core Collection databases was conducted using the following retrieve terms: (Pneumatosis Intestinalis) AND “drug-induced” or “adverse event*” or “adverse reaction*” or “adverse drug reaction*” or “ADR.” In addition, reports assessing ICIs and steroids as suspected drugs for PI were included.

### 3.2 Results

Six studies on steroid-related PI (Galm et al., 2001; Han et al., 2002; Patel et al., 2014; Ozturk et al., 2017; Lee and Wu, 2019; Nunomiya et al., 2021) were included. Patient characteristics, medication therapies, treatments, prognosis, and outcomes are summarized in Table 1. Eight cases of PI secondary to the combined therapy of steroids and other cytotoxic, immunosuppressive agents (Galm et al., 2001; Patel et al., 2014; Ozturk et al., 2017; Nunomiya et al., 2021) were identified, and two cases of PI secondary to steroid monotherapy (Han et al., 2002; Lee and Wu, 2019). In addition, six patients had underlying hematopoietic and lymphoid system conditions (Galm et al., 2001; Patel et al., 2014); one patient had lung adenocarcinoma, another had nasopharyngeal cancer (Ozturk et al., 2017; Nunomiya et al., 2021), and the remaining two had nephrotic syndromes (Han et al., 2002) and superior mesenteric artery syndromes (Lee and Wu, 2019). Symptoms were commonly identified, including abdominal pain and distention, diarrhea, nausea, and vomiting. Free air under the diaphragm, pneumatosis in the intestinal wall, and perforations and gas in other veins were the most reported radiologic findings. In the series report, parenteral nutrition and antibiotics were administered and two cases had associated infections (Galm et al., 2001). Two patients underwent exploratory (Ozturk et al., 2017) and ileostomy surgeries (Patel et al., 2014), while the remaining patients received supportive or conservative treatment. The conservative treatment included oxygen inhalation, metoclopramide, and peritoneal drainage (Han et al., 2002; Lee and Wu, 2019; Nunomiya et al., 2021). It showed that the majority of PI cases occurred within 1 month after suspected drug administration. Most cases were resolved; however, a patient died from respiratory failure (Galm et al., 2001).

**TABLE 1:** The information of patients with pneumatosis intestinalis.

No.	Sex/Age (yr)	Underlying disease	Suspected drug	Dose	Steroid duration (day)	Abdominal symptoms	Radiologic findings	Infection	Location	Treatment	Re-challenge	Outcome
1(Nunomiya et al., 2021)	M/72	Lung adenocarcinoma	Bevacizumab, pemetrexed	Bevacizumab (15 mg/kg), pemetrexed (500	N.A	Asymptomatic	Perforation; pneumatosis in the intestinal wall; no intraportal venous gas	None	Transverse colon	Observation; Supportive care	None	Resolved
				Mg/m^2^)								
2(Ozturk et al., 2017)	M/61	Nasopharyngeal cancer	Docetaxel, fluorouracil	Docetaxel (140 mg), fluorouracil (a total dosage of 7,000 mg)	N.A	Abdominal pain, bilious vomiting	Pneumatosis in the intestinal wall; gas in the portal vein, at the periphery of the liver parenchyma, and in the mesenteric veins	None	N.A.	Exploratory operation; Supportive care	N.A.	Resolved
3(Galm et al., 2001)	M/31	Acute T-lymphoblastic leukemia	Cyclophosphamide, mercaptopurine, cytosine arabinoside, prednisone	N.A.	N.A.	Abdominal pain	Free air under the diaphragm; pneumatosis in the intestinal wall	None	N.A.	Parenteral nutrition and antibiotics	Yes	Recurrence
4(Galm et al., 2001)	F/38	Chronic myelogenous leukemia	Daunorubicin, vincristine, dexamethasone	N.A.	N.A.	Asymptomatic	Free air under the diaphragm; pneumatosis in the intestinal wall; pneumoretroperitoneum; pneumomediastinum	None	Right colon	Parenteral nutrition and antibiotics	None	Resolved
5(Galm et al., 2001)	M/58	Lymphoma	Cyclophosphamide, vincristine, prednisone, dexamethasone, BCNU, etoposide, cytosine arabinoside, melphalan	N.A.	N.A.	Asymptomatic	Free air under the diaphragm; pneumatosis in the intestinal wall	None	The ascending and transverse colon	Parenteral nutrition and antibiotics	N.A.	Resolved
6(Galm et al., 2001)	F/64	Lymphoma	Cyclophosphamide, doxorubicin, vincristine, prednisone	N.A.	10	Diarrhea, abdominal pain and nausea	Pneumatosis in the intestinal wall	Yes	Distal jejunum, proximal ileum, and left hemi-colon	Parenteral nutrition and antibiotics	Yes	Cyclophosphamide was readministered because of the second relapse, no further episodes
7(Galm et al., 2001)	F/49	Aplastic anemia	Ciclosporin A, prednisone	N.A.	N.A.	Fever	Pneumatosis in the intestinal wall, pneumoretroperitoneum, and pneumomediastinum	Yes	N.A.	Parenteral nutrition; antibiotics	None	Died from respiratory failure
8(Patel et al., 2014)	M/14	Lymphoma	Cyclophosphamide, vincristine, daunorubicin, methotrexate, prednisone	N.A.	5	Abdominal pain and distention	Free air under the diaphragm; pneumatosis in the intestinal wall	None	The ascending and transverse colon	Ileostomy	N.A.	Resolved
9(Han et al., 2002)	M/38	Nephrotic syndrome	Prednisone	50 mg daily for 4 months	400+	Abdominal discomfort	Free gas in the retroperitoneal space and mediastinum; pneumatosis in the intestinal wall	None	Right colon	Conservative treatment; oxygen inhalation; metoclopramide	None	Resolved
				60 mg daily for 8 weeks and tapered to 20 mg daily								
10(Lee and Wu, 2019)	M/50	Superior mesenteric artery syndrome, hypersensitivity pneumonitis	Hydrocortisone, dexamethasone, methylprednisolone	Hydrocortisone (100 mg Q8H intravenous 5 days); Dexamethasone (4 mg TID oral 27 days); Methylprednisolone (40 mg BID intravenous 5 days)	37	Abdominal distention and vomiting	Pneumoperitoneum, pneumoretroperitoneum, and pneumatosis in the intestinal wall	None	N.A.	Peritoneal drainage and antibiotics	N.A.	Resolved

## 4 FAERS analysis

### 4.1 Methods

Pneumatosis intestinalis was the preferred term in pharmacovigilance retrieval in the FAERS database, with a time range between the first quarter of 2005 and the third quarter of 2022. Four algorithms, including the reporting odds ratio (ROR), proportional reporting ratio (PRR), information component (IC), and empirical Bayesian geometric mean (EBGM), were used to calculate pharmacovigilance signals (van Puijenbroek et al., 2002). The “*a*,” “*b*,” “*c*” and “*d*” represented case numbers including the suspected drug and the adverse drug reactions (ADRs), case numbers including suspected ADRs with other drugs, case numbers including suspected drug with other ADRs, and case numbers including other drugs and other ADRs, respectively. The equations and criteria for the four algorithms were as follows: ROR=(*a*/*b*)/(*c*/*d*), 95%CI = e^ln(ROR)±1.96(1/*a*+1/*b*+1/*c*+1/*d*)^0.5^, (Criteria:95% CI > 1, *a*≥2); PRR=(*a*/(*a*+*c*))/(*b*/(*b* + *d*)), χ^2^ = Σ((*a*-(*a*+*b*)(*a*+*c*)/(*a*+*b* + *c* + *d*))^2^/((*a*+*b*)(*a*+*c*)/(*a*+*b* + *c* + *d*))) (Criteria: PRR≥2, χ^2^ ≥ 4, *a*≥3); IC = log_2_ *a*(*a*+*b* + *c* + *d*)/((*a*+*c*)(*a*+*b*)), IC025 = e^ln(IC)−1.96(1/*a*+1/*b*+1/*c*+1/*d*)^0.5^ (Criteria: IC025 > 0); EBGM = *a*(*a*+*b* + *c* + *d*)/((*a*+*c*)(*a*+*b*)), EB05 = e^ln(EBGM)−1.64(1/*a*+1/*b*+1/*c*+1/*d*)^0.5^ (Criteria: EB05 ≥ 2, *a*>0).

### 4.2 Results

A total of 1,272 cases of pneumatosis intestinalis related to ICIs (*n* = 62) or steroids (*n* = 1,210) were recorded in the FAERS database between 2005 and 2022. Therapeutic medication included five ICIs (ipilimumab, nivolumab, pembrolizumab, atezolizumab, and avelumab) and six steroids (dexamethasone, prednisone, betamethasone, hydrocortisone, methylprednisolone, and prednisolone). Demographic information, including reporting years and reporters, and patient information regarding age, sex, outcome, and onset time were listed in Table 2. The report numbers and algorithm signals for both groups are listed in Table 3. In ICIs group, the IC signal of the Ipilimumab, and the ROR, the IC, and the EBGM signals of the Nivolumab met the criteria. All four algorithms showed positive signals for the rest drugs in each group. Besides, the FAERS analysis in our research only yielded 138 effective time to onset records and the median time was 92.72 days.

**TABLE 2:** The demography and patient information of ICIs-related or steroids-related pneumatosis intestinalis in FAERS.

Category	Group	ICIs	Steroids	Total
Reporting Year (*n* = 1,272)	2005–2010	0	32	32 (2.52%)
	2011–2013	1	113	114 (8.96%)
	2014–2016	1	107	108 (8.49%)
	2017–2019	20	410	430 (33.81%)
	2020–2022	40	548	588 (46.23%)
Reportor (*n* = 1,272)	Consumer	7	120	127 (9.98%)
	Other health-professional	6	411	417 (32.78%)
	Pharmacist	6	5	11 (0.86%)
	Physician	40	342	382 (30.03%)
	Unknown	3	332	335 (26.34%)
Age (*n* = 1,272)	<18	0	494	494 (38.84%)
	18–44	1	76	77 (6.05%)
	45–64	12	220	232 (18.24%)
	65–74	26	60	86 (6.76%)
	75–84	15	52	67 (5.27%)
	>85	6	13	19 (1.49%)
	Unknown	2	295	297 (23.35%)
Sex (*n* = 1,272)	Female	17	389	406 (31.92%)
	Male	40	452	492 (38.68%)
	Unknown	5	369	374 (29.4%)
Onset time (*n* = 137)	0-10 days	7	38	45 (32.85)
	11-30 days	5	41	46 (33.58%)
	31-240 days	20	20	40 (29.20%)
	>240 days	2	4	6 (4.38%)
Outcome (*n* = 2,256)	congenital anomaly	0	11	11 (0.49%)
	Death	14	540	554 (24.56%)
	Disability	1	1	2 (0.09%)
	hospitalization - initial or prolonged	43	479	522 (23.14%)
	life-threatening	9	111	120 (5.32%)
	other serious (important medical event)	49	997	1,046 (46.37%)
	required intervention to prevent permanent impairment/damage	0	1	1 (0.04%)

**TABLE 3:** Case numbers and detected signals of ICIs-related or steroids-related pneumatosis intestinalis.

Group	Drug	N (%)	ROR (95% CI)	PRR (χ^2^)	IC (IC025)	EBGM (EB05)
ICIs (*n* = 62)	Ipilimumab	7 (0.55%)	1.71 (0.81)	3.59 (1.71)	2.06 (0.77)	0.37 (1.71)
	Nivolumab	21 (1.65%)	2.4 (1.56)	3.68 (2.4)	16.92 (1.25)	0.81 (2.38)
	Pembrolizumab	22 (1.73%)	4.14 (2.72)	6.31 (4.14)	51.88 (2.04)	1.34 (4.11)
	Atezolizumab	10 (0.79%)	3.99 (2.14)	7.42 (3.98)	22.24 (1.99)	1.07 (3.97)
	Avelumab	2 (0.16%)	6.3 (1.57)	25.23 (6.3)	8.91 (2.65)	0.66 (6.29)
Steroids (*n* = 1,210)	Prednisolone	460 (36.16%)	44.36 (39.99)	49.21 (44.16)	15107.04 (5.11)	4.61 (34.6)
	Betamethasone	3 (0.24%)	3.31 (1.07)	10.27 (3.31)	4.82 (1.72)	0.56 (3.3)
	Dexamethasone	151 (11.87%)	13.4 (11.35)	15.81 (13.38)	1,603.61 (3.64)	3.08 (12.48)
	Methylprednisolone	278 (21.86%)	62.52 (55.08)	70.96 (62.07)	14470.07 (5.75)	5.07 (53.9)
	Prednisone	213 (16.75%)	14.57 (12.64)	16.79 (14.55)	2411.41 (3.72)	3.23 (13.16)
	Hydrocortisone	105 (8.25%)	55.3 (45.41)	67.34 (54.92)	5278.47 (5.71)	4.69 (52.2)

N, case numbers; ROR, reporting odds ratio; CI, confidence interval; PRR, proportional reporting ratio; IC, information component; IC025, the lower limit of the 95% two-sided CI of the IC; EBGM, empirical Bayesian geometric mean; EB05, the lower 90% one-sided CI of EBGM.

## 5 Discussion

Reports suggest that PI correlates with drug therapy (particularly prednisone therapy and α-glucosidase inhibitors), chemotherapy, molecular targeted therapy, and immunosuppressive agents (Hisamoto et al., 2006; Shinagare et al., 2012; O'Rafferty et al., 2014). However, the presence of non-specific symptoms increases the likelihood that PI is misdiagnosed or missed in the absence of imaging studies and that current morbidity estimates are inaccurate. Consequently, herein we present the case of a 63-year-old male patient with irAEs who developed PI after prednisone therapy. A literature review and a FAERS database exploration focusing on PI post steroids or ICIs were performed to identify a specific causative agent.

The precise mechanisms leading to PI has yet to be elucidated (Shinagare et al., 2012). The consideration causes are now classified into the following categories. 1) Increased intra-abdominal pressure: factors including intestinal surgery, trauma, colonoscopy, obstruction, tumors, ischemic necrosis, and inflammatory reactions, may increase intraluminal pressure which potentially leads to mucosal dissection (Coriat et al., 2011). 2) Increased intra-pulmonary pressure: increased pressure and alveolar rupture could result into the introduction of air along vascular channels in the mediastinum, tracking downward to the aorta and portal system, and then to the intestinal wall (Azzaroli et al., 2011). 3) Microbial theory: bacteria could penetrate the intestinal wall through increasing the mucosa permeability, decompose nutrients, and produce gas, which leads the development of pneumatosis (Young et al., 1996; Honne et al., 2010). 4) Intestinal mucosal vascular injury: antiangiogenic drug and microangiopathy disrupt the intestinal wall by necrosis of the serosa (Coriat et al., 2011; Chang and Marzan, 2015; Nakagawa et al., 2015).

PI after steroid-containing treatment was observed in patients with acute T-lymphoblastic leukemia, chronic myelogenous leukemia, lymphoma, aplastic anemia, nephrotic syndrome, and superior mesenteric artery syndrome (Table 1). Since numerous clinical conditions are associated with PI, there may be many mechanisms for its development. However, a unified theory has yet to be established for its mechanism (Gazzaniga et al., 2022). A potential mechanism is the immunosuppression by steroids that results in the atrophy of Peyer’s patches, inducing loss of intestinal mucosal integrity and leading to intestinal infection or gas migration (Bhamidipati et al., 2014).

Since the approval of ipilimumab for melanoma treatment in 2011, ICIs have changed the use of therapeutics in solid and hematological malignancies (Schmitt et al., 2022); approximately 50% of patients with malignancies are eligible for ICIs treatment (Haslam and Prasad, 2019). A substantial number of patients treated with ICIs will experience so-called irAEs. The incidence of irAEs in programmed death receptor-1 (PD-1) and PD-L1 inhibitors is approximately 15%, while in cytotoxic T lymphocyte antigen-4 (CTLA-4) antibody therapy is approximately 35%, and in the combination of CTLA-4 and PD-1 antibodies is approximately 55%. (Arnaud-Coffin et al., 2019). Although nuanced and targeted treatment of irAEs is desirable, for most moderate-to-severe irAEs, guidelines recommend the initial use of steroids (Haanen et al., 2018). Therefore, the potential risk of steriods-related PI warrants further study.

In general, there is a clear gender difference in hormone-related adverse reactions, and similar disproportion were found in our study. Both the FAERS database and case reports indicate that male appear to be more susceptible to drug-induced PI than female. Interestingly, gender difference was not limited to the steroids. Several studies have indicated that biological differences in sex hormones, body composition, and glucose metabolism may contribute to the disparity. Nevertheless, gender difference in drug-related PI requires further investigation.

Suspected sintilimab-induced PI should not be excluded, although studies reporting this finding have yet to be published. Sintilimab is a domestic PD-1 inhibitor in China that was approved for squamous and non-squamous non-small cell lung cancer by the National Medical Products Administration (Zhang et al., 2022). The gastrointestinal tract is commonly affected by ICIs (Rajha et al., 2020). However, normal bowel movement was observed in our patient during the six cycles of treatment with sintilimab. This suggests that PI likely correlated with methylprednisolone administration.

Oral steroid preparations tend to be highly bioequivalent (Francisco et al., 1984). The systemic bioavailability of prednisone and prednisolone are similar. Varying preparations of methylprednisolone also tend to be bioequivalent, although their oral and rectal absorption is uneven, in a relative bioavailability range from 50% to 90% (Garg et al., 1979). The pharmacokinetics of steroids in diseases and pathophysiological conditions, including severe liver disease, cystic fibrosis, end-stage kidney disease, hemodialysis, nephrotic syndrome, hyperthyroidism, obesity, and pregnancy, are diverse. In our case report, the patient had none of the above-mentioned conditions or off-label medication usage.

The diagnosis of PI mainly relies on imaging and endoscopy, which might easily lead to misdiagnosis and missed diagnosis because of the low incidence and non-specific clinical manifestations. Clinicians should pay attention to PI, collect medical history in detail, and analyze carefully. When imaging examination reveals free gas in the abdominal cavity but lacks symptoms of peritoneal irritation, PI should be considered as a possibility in order to diagnose and treat patients more rationally and avoid unnecessary surgical procedures.

Conservative treatments for PI, including administering oxygen at high concentrations, fasting, and antibiotics, are recommended for individuals with clinical manifestations of the condition (Feuerstein et al., 2014). CT scans are more sensitive to the accurate diagnosis of PI than plain radiographs, increasing the potential for identifying life-threatening conditions (Di Pietropaolo et al., 2020). PI without evidence of further intra-abdominal pathology does not necessitate laparotomy (Galm et al., 2001). PI complicated by bowel obstruction or ischemia tends to require emergency surgical intervention, which correlates with a higher clinical severity score (including degrees of pain, fever, tenderness, diarrhea, blood *per rectum*, and hypotension) (Yang et al., 2022). In our case, the absence of peritonitis, ischemia, and perforation, enabled conservative treatment with ceftriaxone, omeprazole, and sandostatin. Complete resolution of the PI was achieved following prednisone decrement and conservative therapy. However, this resolution should not preclude putting patients on the surgical alert list as the patients are still at risk for perforations and ischemia.

To our knowledge, this is the first study to evaluate irAE treatment-related PI. The study also compared the onset of PI secondary to different steroids in studies published and in the FAERS database. Therefore, this case report emphasizes the potential adverse events of PI associated with steroid use in the management of irAE. The onset of PI as an adverse event from steroids use requires further investigation.

## Data Availability

The original contributions presented in the study are included in the article/supplementary material, further inquiries can be directed to the corresponding authors.

## Abbreviations

Pneumatosis intestinalis, (PI); immune-related adverse event, (irAE); immune checkpoint inhibitor, (ICI); FDA Adverse Event Reporting System, (FAERS); computed tomography (CT); programmed cell death 1 ligand 1, |(PD-L1); reporting odds ratio, (ROR); proportional reporting ratio, (PRR); information component, (IC); empirical Bayesian geometric mean, (EBGM); adverse drug reaction, (ADR); confidence interval, (CI); cytotoxic T lymphocyte antigen-4, (CTLA-4); programmed death receptor-1, (PD-1).
